# Precocious puberty in male wild boars: a possible explanation for the dramatic population increase in Germany and Europe

**DOI:** 10.7717/peerj.11798

**Published:** 2021-07-20

**Authors:** Claudia Maistrelli, Hanna Hüneke, Marion Langeheine, Oliver Keuling, Ursula Siebert, Ralph Brehm

**Affiliations:** 1 Institute for Terrestrial and Aquatic Wildlife Research, University of Veterinary Medicine Hannover Foundation, Hannover, Germany; 2 Institute for Anatomy, University of Veterinary Medicine Hannover Foundation, Hannover, Germany

**Keywords:** Androgen receptor, Anti-Müllerian hormone, Male reproduction, Precocity, Puberty, Spermatogenesis, *Sus scrofa*

## Abstract

**Background:**

The wild boar population in Europe is steadily growing, one of the reasons for this increase probably being the high reproductive potential of this large mammal. Population management is important to stabilise wild boar numbers and a great deal of attention is focusing on the reasons, which might contribute to the high reproductive rates. Understanding the timing of puberty attainment provides information required for proper management practices. Knowledge of the earliest expected time of sexual maturation in male wild boars is limited, research being mostly focused on females. Previous hunting references indicate that sexual maturity in males occurs in the second year after birth. In contrast, male domestic pigs become sexually mature from about seven months of age. Thus, aims of this study were to investigate (1) whether there is a physiological ability for reproduction also in male wild boars of a younger age and (2) whether the body weight of wild boar males has a more important role than age in driving the maturation of the testis.

**Methods:**

Male wild boar individuals were sampled during hunting drives in the eastern part of Lower Saxony in Germany. Testes with epididymides from 74 males were collected and prepared for histological examination and immunohistochemistry. The reproductive status could be ascertained based on development/occurrence of different germ cell populations using histology and based on the immunohistochemical detection of the anti-Müllerian hormone and androgen receptor.

**Results:**

In this study, male wild boars aged nine to ten months already passed puberty and were able to reproduce if they had reached the appropriate body condition of about 29 kg dressed weight. Immunopositivity to the anti-Müllerian hormone in Sertoli cells was evident only in prepubertal animals and decreased with the onset of puberty. No immunoreaction was evident at postpuberty. The androgen receptor was detected in Sertoli cells, peritubular cells and Leydig cells, surprisingly already in Sertoli cells of prepubertal wild boars as well depending on body weight. Moreover, two-thirds of young males aged about ten months were precociously reproductively mature, showing histologically the presence of spermatozoa in testes and epididymides.

**Conclusions:**

As piglets are mostly born in spring, also these young male individuals could target the heat of female wild boars in the winter months, resulting in the observed population increase. Therefore, a reduction in wild boar numbers should also focus on piglets of both sexes.

## Introduction

In Europe, since some decades, populations of European wild boar (*Sus scrofa*) are continuously growing in numbers (Massei et al., 2014). Despite the fact that the wild boar has high commercial potential because of its meat, this dramatic increase in its population, e.g., in Lower Saxony (Gräber et al., 2018), representing other regions of Germany, has arisen conflicts regarding agriculture and farming as well as environmental balance. Moreover, wild boar could be pestiferous to domestic pig populations and may transfer diseases, e.g., African swine fever, in an uncontrollable manner (Costard et al., 2013).

One of the reasons which explains the growing population trend is the high reproductive rate of this wild ungulate (Massei et al., 2014) as well as the ability for young wild boars to take part in reproduction and so contributing to the high observed animal numbers (Gethöffer, Sodeikat & Pohlmeyer, 2007). An important aspect in reproduction research is that regarding the possible onset of puberty. Studies about attainment of puberty in young wild boars focused mainly on females (Gaillard, Brandt & Jullien, 1993; e.g., Gethöffer, Sodeikat & Pohlmeyer, 2007; Cellina, 2008; Fonseca et al., 2011), whereas to the best of our knowledge, only single publications investigated puberty in the males. The domestic pig, of which the wild boar is ancestor, reaches puberty at about five months of age and thereafter sexual maturation from about seven to nine months after birth, depending on the breed (Andersson et al., 1998; Cheon et al., 2002; França, Avelar & Almeida, 2005; Fraser et al., 2016; Willam & Simianer, 2017). In contrast, common hunting references assumed puberty in the male to occur in the second year after birth (Briedermann, 1990). Only recently it was reported that wild boar males younger than one year might already be able to reproduce (Drimaj et al., 2019).

Thus, it is evident that there exists a lack of basic knowledge about the exact time period at which puberty and consequently sexual maturity occur in the male wild boar. The newly literature suggested more precocious onset of puberty and, therefore, the ability for male wild boars aged one year and/or even younger to reproduce, may also be responsible for the increasing populations of wild boar in Germany and other European countries. Hence, better understanding the timing of puberty attainment can provide useful information for proper management of this species, also in order to manage efficiently conflicts between humans and wild boar.

With this background in mind, in the present study, we aimed to determine when wild boar males reached puberty and could be able to reproduce. We performed histological investigations and immunohistochemistry of the anti-Müllerian hormone and the androgen receptor of testes collected from hunted wild boars of a population located in Lower Saxony, Germany. We assessed puberty by observing the stage of germ cell development during the spermatogenic process, as one of the markers of puberty onset is the beginning of spermatogenesis (Glass, Herbert & Anderson, 1986; Guimarães et al., 2013), which leads, in mature testis, to the development from spermatogonia to elongated spermatids and ultimately spermatozoa (Almeida, Leal & França, 2006; Huang et al., 2011).

The pubertal attainment is also associated with changes in the expression of the anti-Müllerian hormone and the androgen receptor on different cells in the testis (Ramesh et al., 2007; Almeida et al., 2012). A positive detection with immunohistochemistry of the anti-Müllerian hormone connotes animals in which puberty has not been reached yet, since this hormone is secreted by somatic Sertoli cells only until they stop to proliferate and undergo maturation, a process regulated by increasing testosterone levels at puberty onset (França, Avelar & Almeida, 2005; Ford & Wise, 2009; Rey et al., 2009; Kao et al., 2012). As a result of rising testosterone concentrations, the expression of the androgen receptor in Sertoli cells within the seminiferous epithelium, representing the binding site for androgens (Pearl, Mason & Roser, 2011), is increased and marked by a positive reaction at immunohistochemistry.

Therefore, based on the presence (or absence) of specific germ cell population and based on the immunohistochemical results, we could indicate whether an animal had reached puberty or not. We hypothesized that wild boar males reached puberty at a similar age as the domestic pig, already in the first year of age, and so much earlier than common literature suggests. We also supposed that the body weight could drive the attainment of puberty much more efficient than a specific required age, as with regard to evidence in the human medicine (Blum et al., 1997; Aydin et al., 2017), adequate body energy reserves are essential to continue the reproductive development until sexual maturation.

## Materials & Methods

### Samples

In total, testes and epididymides of 74 free-ranging hunted wild boar males were sampled during two hunting seasons, between November 2011 and January 2012 (*n* = 34) and between October 2012 and January 2013 (*n* = 40), respectively (Table 1). Permission for collection of samples was provided verbally by the following heads of operations: Mr. Helmut Beuke (Forest Commission Oerrel), Mr. Georg Deeken (Forest Commission Unterlu ÿ) and Gunther Graf von der Schulenburg (Graf von der Schulenburg). No approval was needed for involving hunted free-range vertebrate animals (wild boars) due to the German law for animal welfare.

All age classes were well represented during these hunting bags. Animals older than two years were also included in the sampling collection as controls. Sample collection and age classification occurred before gutting the carcass. Both testes with epididymides were collected of each individual and prepared on the same day for further histological/immunohistochemical examinations. Age classification was performed for each animal by teeth examination of the lower jaw in accordance with Briedermann (1990) and Heck & Raschke (1980), which allows age estimation monthly up to three years of age. Sampled animals were then weighed after all internal organs had been removed (dressed weight).

### Study area

**Figure 1:**
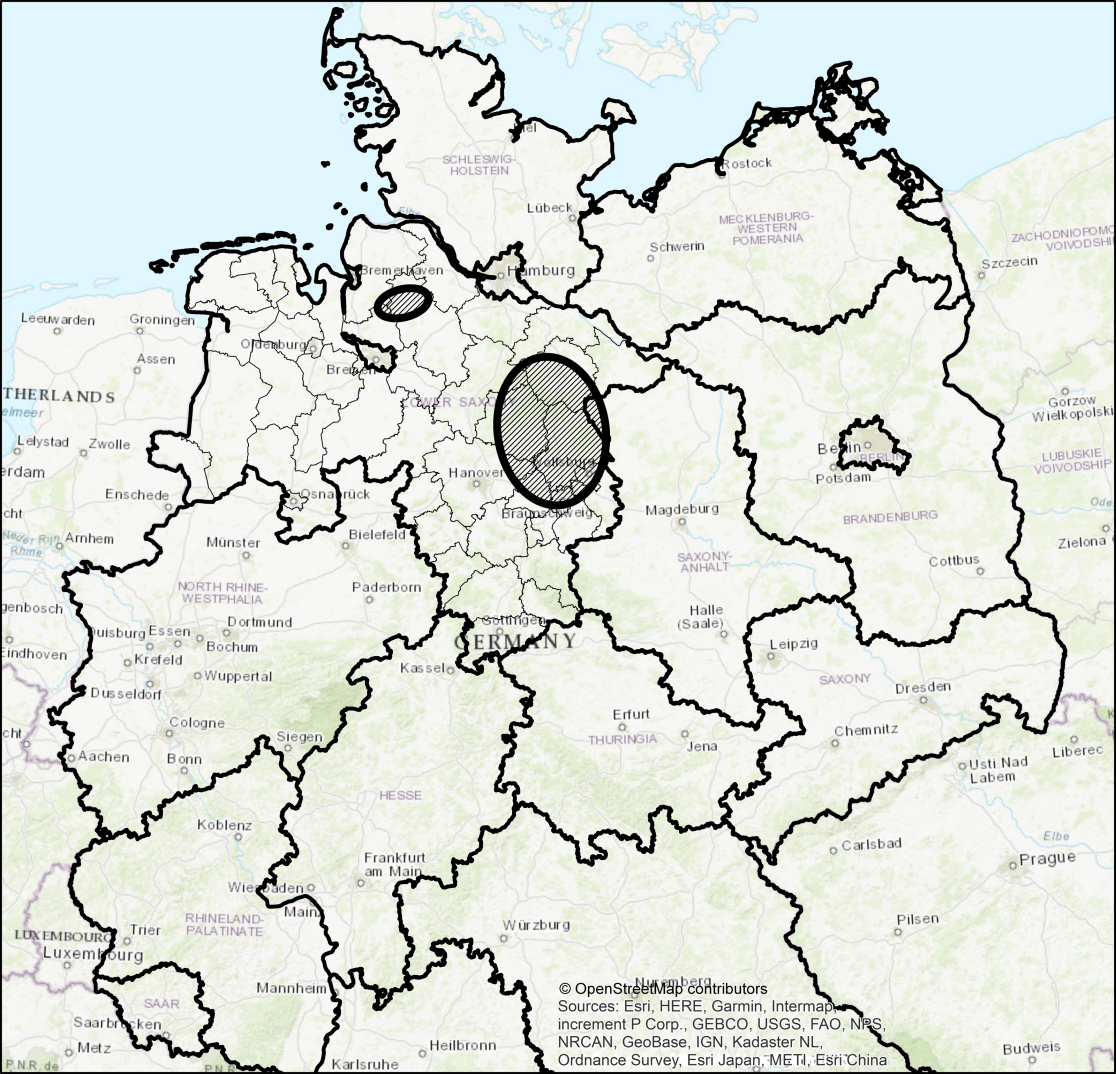
Study area in Lower Saxony, Germany. Study areas are shown as circles with diagonal lines. Thick contour lines delimit German federal state borders. Districts of Lower Saxony are marked with thin lines. Map data ©OpenStreetMap contributors.

Samples were collected during hunting drives which took place on different hunting grounds of the Forestry Commissions Wolfenbüttel, Unterlüß and Graf von der Schulenburg in Lower Saxony, Germany. The Forestry Commissions Wolfenbüttel and Unterlüß manage around 16,400 ha (and 9,448 ha of private forest) and 18,500 ha of forest and both Forestry Commissions are situated in the southeastern part of the Lüneburger Heath and in the Weser-Aller plains. The Forestry Commission Graf von der Schulenburg manages about 4,900 ha of forest in southeastern Lower Saxony (Fig. 1). All study areas are, in general, characterised by a similar temperate moderate climate and about 40% of forest coverage (mixed woodland of mainly pine, spruce, beech and oak) (Dammann et al., 2012).

**Table 1:** Sample collection. Total number of samples and their relative amount for each sexual maturity class and location.

N of samples	Samples/sexual maturity class			Forestry Commission
	**Prepubertal**	**Pubertal**	**Postpubertal**	
20	4	3	13	Wolfenbüttel
27	7	3	17	Unterlüß
27	8	6	13	Graf von der Schulenburg

### Histological examination of the testes

Each testis of the sampled wild boar males was cut into two pieces 0.5 cm × 1 cm × 1 cm, which were then put in Bouin’s solution to fix for the subsequent histological and immunohistochemical studies. Prepared tissue was then embedded in paraffin and sliced into 4 µm sections using a rotating microtome. After drying, the sections for histological examinations were stained with haematoxylin-eosin and examined under a light microscope. The obtained testis sections were investigated regarding the different developmental stages of germ cells in the seminiferous cords or tubules (gonocytes, spermatogonia, spermatocytes, round, and elongated spermatids), whereas sections of epididymides were investigated regarding the presence or absence of spermatozoa. Based on the progress of spermatogenesis, testis samples were classified as either (1) pubertal, still (2) prepubertal or already (3) postpubertal. Briefly, sections showing Sertoli cells and germ cells developing until spermatocytes/round spermatids formed within the seminiferous epithelium and absence of sperm in the epididymides were classified as pubertal. The sections of seminiferous cords in which spermatogenesis was not started yet, presenting only gonocytes, spermatogonia and somatic Sertoli cells as well as empty epididymides were defined as prepubertal. Testis sections characterized by qualitatively and quantitatively normal, complete spermatogenesis, i.e., by the presence of elongated spermatids and spermatozoa in the seminiferous tubules as well as by the presence of spermatozoa in the epididymis were considered postpubertal.

### Immunohistochemistry

After deparaffinisation and inhibition of the endogenous peroxidase activity with 3% H2O2 in 80% ethanol for 30 min, sections for androgen receptor immunohistochemistry were microwave pretreated with sodium citrate buffer (pH 6.0) for 3 × 5 min at 800 W (anti-Müllerian hormone sections for 20 min at 96−99 °C on a heating plate). Slides were then cooled down for 30 min at room temperature, blocked with 3% bovine serum albumin for 20 min and incubated with the respective primary antibody (anti-AR, Santa Cruz Biotechnology, Inc., Dallas, TX, USA, Catalogue No.: sc-816, dilution 1:250) over night at 4 °C. The sections for androgen receptor were then exposed for 30 min at room temperature with EnVisionTM +Kit HRP Rabbit DAB+ (Dako Deutschland GmbH, Hamburg, Germany, Catalogue No. K4011), sections for anti-Müllerian hormone immunohistochemistry (anti-AMH, Abcam PLC, Cambridge, UK, Catalogue No.: ab24542, dilution 1:50) were treated with EnVisionTM +Kit HRP Mouse DAB+ (Dako Deutschland GmbH, Hamburg, Germany, Catalogue Catalogue No. 4007) for 30 min at room temperature. After visualisation with DAB, sections were counterstained with haematoxylin for 4 s and rinsed with running water. Finally, all slides were mounted with Eukitt™ (O. Kindler GmbH, Freiburg, Germany). Negative controls were performed by substituting the primary antibody with buffer. Stained sections were examined under a light microscope to assess the puberty onset within the seminiferous epithelium through the (gain of) expression of the androgen receptor and (the loss of) the anti-Müllerian hormone in Sertoli cells.

### Statistical analysis

All statistical analyses were conducted with RStudio version 1.2.5019. Data were presented as the mean ± SD. Analysis was performed with moderndive (ver.0.4.0) and tidyverse (ver.1.2.1) packages and data visualisation with the package ggplot2 (ver.3.2.1) (Kassambara, 2013). Homogeneity of variances between groups for both variables age and dressed weight were tested with Levene’s test. Anova and Tuckey’s HSD (honestly significant difference) were performed to compare differences between and within groups for both variables age and dressed weight. Differences between study areas and sampling seasons were tested with Student’s *t*-test. A generalised linear mixed model was used to examine the probability of sexual maturity onset and interactions between the variables, age and dressed weight. These two prediction variables showed multicollinearity with a VIF (variance inflation factor) greater than five. Indeed, age and weight were allometric variables regarding the age (which was increasing), i.e., inevitably in strong relationship. Due to this and after analysis of AIC, i.e., the choice of the model with lowest AIC and therefore that which had the most parsimonious fit, the variables age and weight were modelled separately. Furthermore, to obtain a binary response variable, samples were divided into two categories only. Thus, the prepubertal and the pubertal classes were grouped together. This was performed with the package car (ver.3.0-7) and effects (ver.4.1-4).

A *p*-value <0.05 was considered to be statistically significant.

## Results

### Histology and characteristics of wild boar males

**Figure 2:**
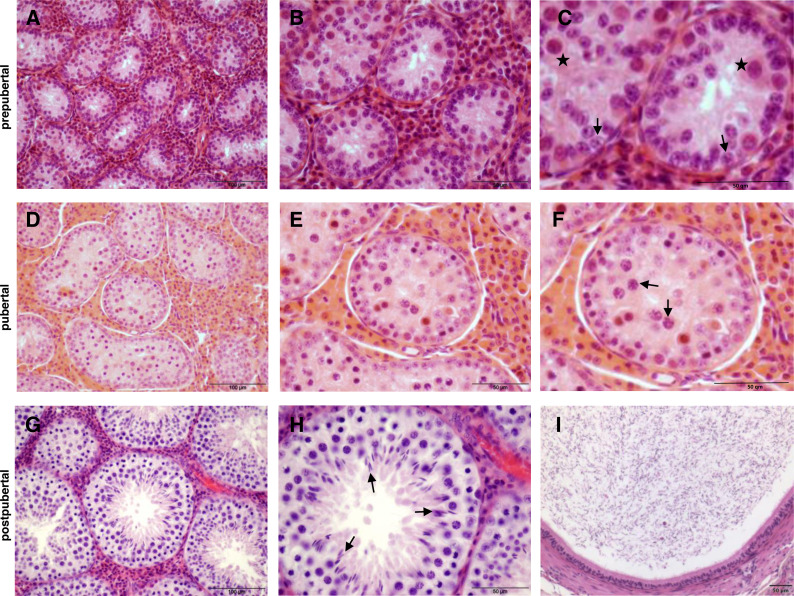
Histology of wild boar testes and epididymides after haematoxylin-eosin staining. (A, B, C) Prepubertal: presence of Sertoli cells, gonocytes (stars in C) and spermatogonia (arrows in C). (D, E, F) Pubertal: presence of spermatocytes (arrows in F). (G, H, I) Postpubertal: normal spermatogenesis, presence of elongated spermatids (arrows in H) and presence of spermatozoa in epididymis (I). Scale bars: 100 µm (A, D, G); 50 µm (B, C, E, F, H, I).

The histological investigations which served to classify the 74 collected wild boar testes based on the progress of spermatogenesis are shown with representative sections examples in Fig. 2.

The characteristics of the sampled wild boar males expressed as mean age (in months) and mean weight (in kg) are presented in Table 2. Male wild boars (*n* = 13) which were more than 18 months of age and/or 50 kg of dressed weight were not relevant for the purpose of this study and were not further evaluated, as a more advanced age or heavier weight connotes adulthood (Table 2).

**Table 2:** Characteristics of wild boar males. Data are presented as mean ± SD.

**Sexual maturity class**	**Animals (n)**	**Mean age (months)**	**Mean weight (kg)**
1=Prepubertal	19	8.5 ± 1.2	23.2 ± 5.0
2=Pubertal	12	9.3 ± 1.2	29.6 ± 3.6
3=Postpubertal	27 (43)^*^	10 ± 0.8 (14.5 ± 7.1)^*^	34.6 ± 6.4 (43.2 ± 1.6)^*^

**Notes.**

* Total number and mean age and weight of postpubertal animals including >18 months and >50 kg.

**Figure 3:**
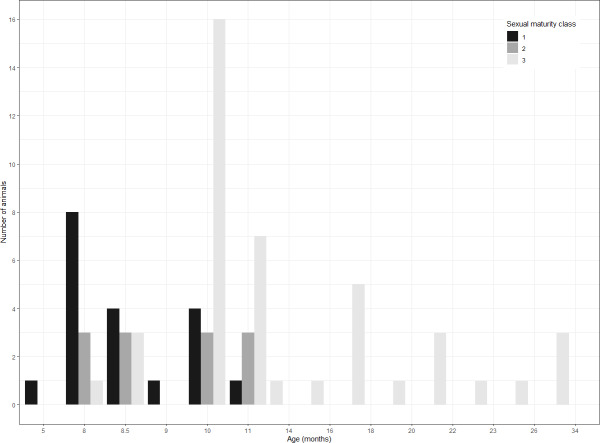
Distribution of sampled wild boar males. Age is expressed in months. Sexual maturity class: prepubertal (1), pubertal (2), postpubertal (3).

**Figure 4:**
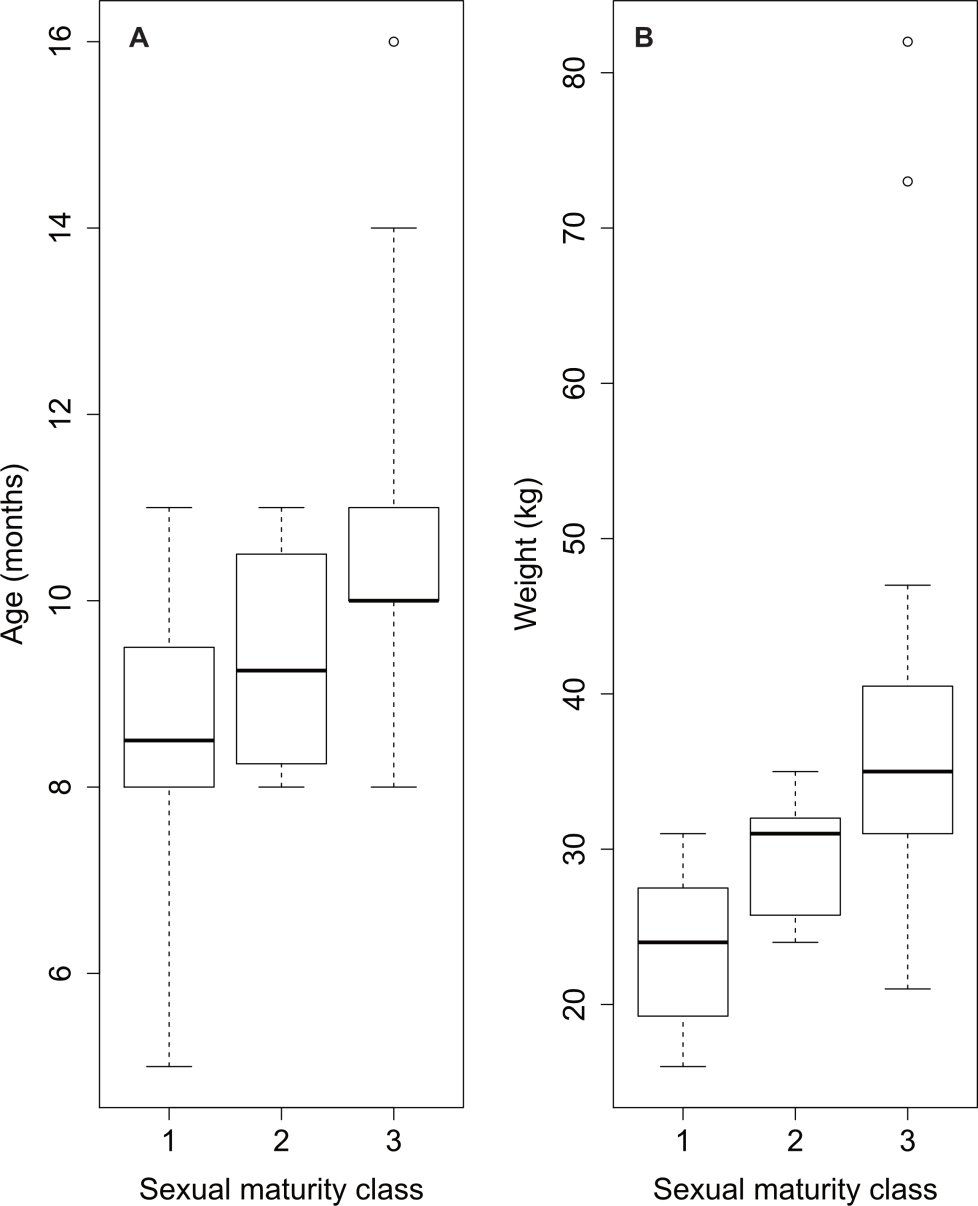
Overview of sexual maturity classification of sampled wild boar males based on age (A) and on weight data (B). (A) Classification depending on age shows pubertal class (2) overlapping classes 1 and 3; (B) classification depending on dressed weight shows more variance in the data.

The distribution of the three classes across age is shown in Fig. 3. The prepubertal and the postpubertal stages overlapped between the eighth and the eleventh month of age, whereas there was more variance when considering the variable dressed weight (Fig. 4). Indeed, Levene’s test showed a significant difference in the variances for the variable weight (*p*-value = .003) that was greater than for the variable age. This was further confirmed by the analysis of variance that revealed a significant difference in the mean dressed weights of the sexual maturity classes (*p*-value = .00001). Tukey’s procedure for pairwise comparisons of prepubertal, pubertal and postpubertal males showed significant differences between all pairs of means regarding the dressed weight. The difference in the mean ages was less significant and reported just between class 1 (prepubertal) and class 3 (postpubertal) (*p*-value = .00072).

Student’s *t* test used to test differences between sampling areas and sampling seasons for the variables age and weight in the prepubertal and pubertal classes, respectively, did not show any significant differences neither between sampling areas nor between the sampling seasons.

### Immunohistochemistry

**Figure 5:**
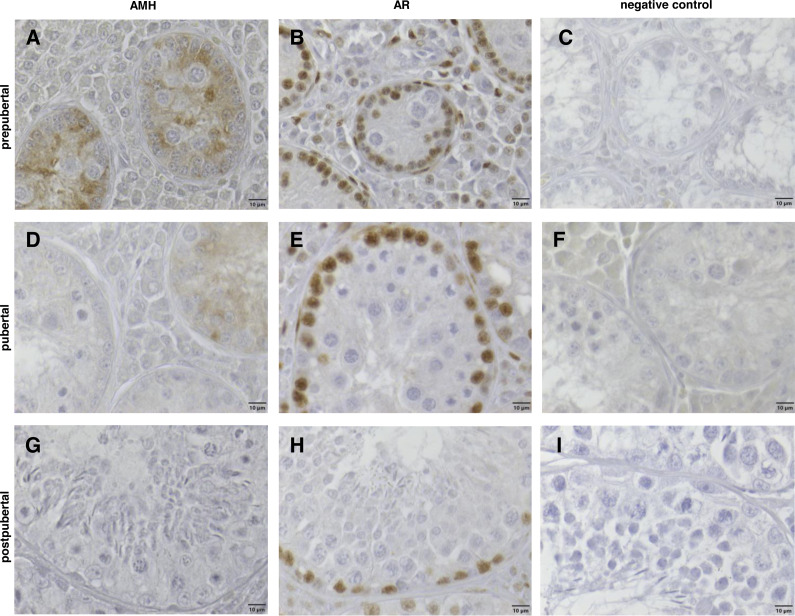
Immunhistochemical stainings of anti-Muellerian-Hormone (AMH) and of Androgen Receptor (AR) from prepubertal (8 months/20 kg), pubertal (11 months/31 kg) and postpubertal (10 months/34 kg) wild boar testes counterstained with haematoxylin. (A, D, G) AMH immunohistochemistry: from prepubertal (A) to pubertal (D) age, the cytoplasmic staining of Sertoli cells (SC) regressed; in postpubertal (G) SC, no AMH immunopositive reaction was detectable. (B, E, H) AR immunohistochemistry: markedly positive nuclear staining of SC, peritubular cells and Leydig cells were visible. All nuclei of the different germ cell populations were immunonegative. (C, F, I) Respective negative controls. Scale bars: 10 µm.

Anti-Müllerian hormone was immunohistochemically detected in the cytoplasm of somatic Sertoli cells from prepubertal male wild boars only (Fig. 5A). It was reduced in pubertal samples relating to the onset of puberty (Fig. 5D), and in postpubertal wild boars it was no longer detectable (Fig. 5G). The anti-Müllerian hormone was only detected within the seminiferous epithelium in Sertoli cells and not in germ cells.

Positive nuclear staining of Sertoli cells, peritubular cells and Leydig cells was visible for the androgen receptor in every sexual maturity class, also in the prepubertal one depending on body weight. All nuclei of the different germ cell populations were immunonegative (Figs. 5B, 5E, 5H). All samples in which the primary antibody had been substituted with buffer (control group) were negative (Figs. 5C, 5F, 5I).

**Figure 6:**
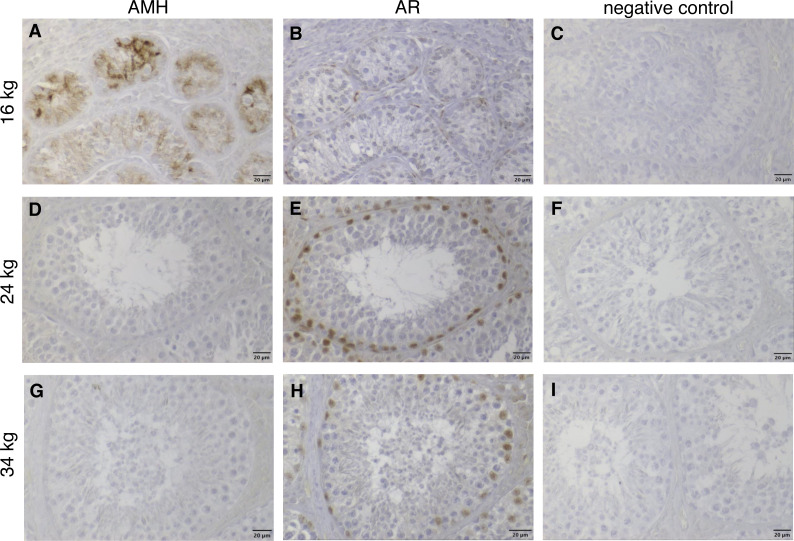
Immunhistochemical stainings of anti-Muellerian-Hormone (AMH) and of Androgen Receptor (AR) in testes of three 10-months-old wild boars with different dressed weights (16, 24 and 34 kg), counterstained with haematoxylin. The three selected wild boar males were of same age (10 months), but showed totally different stages of spermatogenesis and dressed weights. (A, D, G) AMH immunohistochemistry: the 16 kg wild boar of 10 months of age with cytoplasmic staining in Sertoli cells (SC) characterized by prepubertal development (A), a 24 kg pubertal (D) and a 34 kg postpubertal (with visible complete spermatogenesis) (G) wild boar of same age as A, but with immunonegative AMH reaction. The dominant role of body weight over age is evident. (B, E, H) AR immunohistochemistry: markedly positive nuclear staining of SC (E, H), peritubular cells (B, E, H) were visible. All nuclei of the different germ cell populations were immunonegative. (C, F, I) Respective negative controls. Scale bars: 20 µm.

In addition, a comparison between wild boar males showing different developmental stages and spermatogenesis (belonging, respectively, to the three sexual maturity classes) but being all of same age was performed using histology and immunohistochemistry. These animals were ten months old, however, differed in their dressed weights, remarking the reported variance of weight observed above. In detail, they were two (out of four) ten-month-old prepubertal males (Fig. 3) weighing 16 and 19 kg, respectively, one pubertal wild boar weighing 24 kg as well as one postpubertal male that weighed 34 kg. At immunohistochemical investigation (Fig. 6) it was possible to confirm our hypothesis. Briefly, the prepubertal male with 16 kg dressed weight showed positive immunostaining for the anti-Müllerian hormone (Fig. 6A) as well weak positive reaction to the androgen receptor just in peritubular cells (Fig. 6B). In contrast, in the heavier animals, namely in the afore mentioned pubertal (Figs. 6D, 6E) and postpubertal wild boars (Figs. 6G, 6H), the anti-Müllerian hormone was not more detectable whereas the staining for the androgen receptor was now also strongly positive in somatic Sertoli cells.

### Onset of sexual maturity

**Figure 7:**
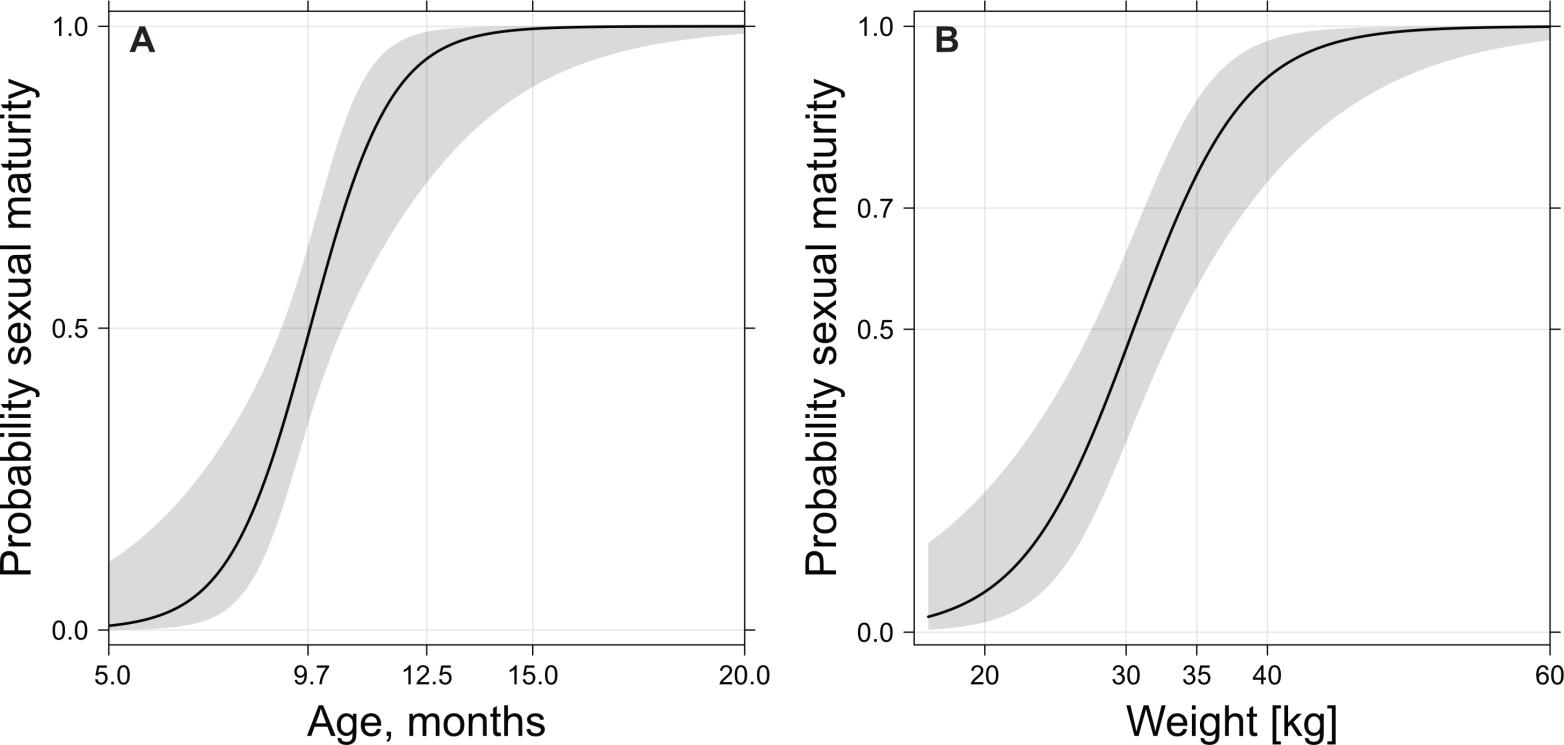
Probability of sexual maturity onset depending on age (A) or weight (B) after grouping together prepubertal/pubertal and postpubertal classes. Age is expressed in months and dressed weight in kg. From nine to ten months of age or 30 to 35 kg dressed weight, the probability of sexual maturity onset increased from about 50 to 70%.

Dressed weight was also a better variable than age in modelling the probability of reaching sexual maturity. The probability of reaching sexual maturity (Fig. 7) increased from about 50 to about 70% between 30 and 35 kg dressed weight (Fig. 7A), which corresponded to an age of nine to ten months (Fig. 7B). Almost all investigated wild boar males completed spermatogenesis with a dressed weight of 40 kg.

## Discussion

Knowledge about the most precocious onset of puberty and attainment of sexual maturity plays a key role in assessing the reproductive cycle and reproductive potential of a population (Kesselring et al., 2017), which, in turn, is a determinant for an efficient population management. Puberty is commonly described for male individuals as the completion of the first wave of spermatogenesis, which leads to the onset of sexual activity and the ability to take part in reproduction (Tauson, 2012). In the present study, we first performed a histological investigation of testes and epididymides in accordance with literature sources (e.g., Ford & Wise, 2009; Ren et al., 2009; Ford & Wise, 2011; Howroyd, Peter & De Rijk, 2016; Kangawa et al., 2016; Taberner et al., 2016; Sarnklong et al., 2020). Moreover, for the first time to our knowledge, we then performed the immunohistochemistry for the anti-Müllerian hormone and the androgen receptor in the male wild boar testis. Thus, combined with the histological analysis, we could objectively assess the attainment of puberty as well as the onset of sexual maturity.

In general, more than one parameter should be considered while assessing when an animal becomes mature, i.e., is able to mate and produce a sufficient number of viable gametes (Howroyd, Peter & Rijk, 2016). However, in the literature, we found several definitions used as parameters to assess when an animal can be considered pubertal and then able to reproduce (Howroyd, Peter & De Rijk, 2016; Kangawa et al., 2016), this probably because the onset of puberty is often defined by species-specific hallmarks of pubertal development (Smeets, 2015). For example, studies have determined the achievement of puberty with analysis on the ejaculate of pigs and have considered those individuals as pubertal who had normal spermatozoa in the cauda of the epididymis (Karunakaran et al., 2009), in other cases, the parameter used for wild boars was the presence of visible ducts in the cauda epididymis (Ahmad et al., 1995) or the presence of sperm combined to an increase in plasma testosterone concentrations (Mauget & Pepin, 1991). Recently, a study on micropigs determined histologically the achievement of puberty based on the presence of active spermatogenesis in the seminiferous tubules and the first spermatozoa in the cauda epididymis, whereas sexual maturity was then reached shortly thereafter, when spermatogenesis was complete and the other reproductive organs reached full development (Kangawa et al., 2016).

In contrast to the above reported studies, we considered animals as pubertal those that presented spermatocytes and round spermatids as the most advanced germ cell type in the seminiferous tubule. The absence of sperm in the epididymis was a further indication thereof, which was confirmed by histology and the immunohistochemical investigations using testicular sections. In fact, the attainment of puberty was characterised by a decreased immunopositivity to the anti-Müllerian hormone in Sertoli cells until complete loss of detection at postpuberty. The presence of spermatozoa was observed only in postpubertal animals which consequently did not show any positive reaction to the anti-Müllerian hormone. According to the literature, the anti-Müllerian hormone is no longer secreted by somatic Sertoli cells that have undergone maturation at the beginning of pubertal development (Edelsztein & Rey, 2019), this therefore being a useful hallmark of puberty attainment also for the wild boar male.

In more recent years, puberty onset was assessed in the wild boar already at six months of age (Drimaj et al., 2019). In this study, wild boar males showing presence of sperm in imprinting preparations of epididymides were considered pubertal and sexually mature (Drimaj et al., 2019).

In our study, instead, puberty was attained at about nine months of age and 29 kg body weight and the onset of sexual maturity was reached on average at about ten months of age and 34 kg. The observed differences may depend on diverse age determination according to cited references, which could have led to a lower age estimation in comparison to ours. Nevertheless, divergences depend, again, on different criteria used to assess puberty and sexual maturity, which in contrast to our study were used interchangeably and were not well defined and on the different methods applied.

Our results deviate from information on puberty attainment in the second year of age provided by common hunting literature (Brehm, 1922; Briedermann, 1971; Briedermann, 1990), but are more similar to findings in the few previous studies on male wild boars (Mauget & Boissin, 1987; Mauget & Pepin, 1991) or to investigations performed on domestic pig breeds, in which puberty and the onset of sexual maturity occurred at about five and seven months of age, respectively (França et al., 2000; França, Avelar & Almeida, 2005; Avelar et al., 2010; Taberner et al., 2016). Thus, we were able to support our hypothesis that wild boar males reached puberty at a younger age, which is similar to the domestic pig.

In the present study, moreover, puberty was a rather short transitional phase, with the prepubertal and the postpubertal stages overlapping between the eighth and the eleventh month of age. This was evident from the little variation between the mean age of prepubertal and the mean age of pubertal animals. Instead, more variance was represented by the body weight. In fact, besides genotype, environment and season, management, and physiological determinants (Foster, Yellon & Olster, 1985; Chenoweth & Lorton, 2014; Gobello, 2014), body energy reserves are one major factor contributing to normal onset and progress of puberty. Adequate nutrition is known from human medicine to play a permissive role in puberty attainment; undernutrition or poor body condition such as extreme low adiposity causes delay in puberty onset (Aydin et al., 2017). Our data confirmed the hypothesis that body weight had a permissive role, besides age, for puberty onset, which is also similar to previous studies that assessed a relationship between age and body weight for the attainment of sexual maturity (Pépin et al., 1987; Mauget & Pepin, 1991) or to those studies which accentuated the importance of body weight over age (Drimaj et al., 2019). The permissive role of body weight was especially evident in two prepubertal individuals: even if they attained a pubertal age of ten months and were therefore older than the average age in the prepubertal class, they still showed an immature development of the testes. Indeed, they presented a poor body condition with dressed weights far below 29 kg, weighing only 16 and 19 kg, which is a surprisingly light dressed weight in comparison to their age. In contrast, other males aged ten months, but presenting heavier weights, already reached puberty or even presented a complete spermatogenesis. Good body condition clearly influenced the onset of puberty, anyway, in those males weighing under 30 kg of body weight that attained postpuberty, age still played a determinant factor in sexual development, since these animals were at least ten months old.

In conclusion, regarding the immunohistochemical detection of the androgen receptor in Sertoli cells, we observed (strong) positivity in every sexual maturity class depending on the weight. Surprisingly, we did not observe a clear inverse relationship between the anti-Müllerian hormone and the androgen receptor depending on the stage of pubertal development (Rey et al., 2009; Edelsztein & Rey, 2019). In prepubertal individuals, testes are already capable of testosterone production with its concentrations increasing gradually, which serves as a main trigger for Sertoli cell maturation, downregulation of the anti-Müllerian hormone and completion of full spermatogenesis (Rey et al., 2009; Rey, 2021) as well as for development of normal, mature, external male phenotype (Wang et al., 2009). In fact, Sertoli cell population stabilises during the prepubertal period and their maturation between the prepubertal and postpubertal period coincides with the appearance of first spermatocytes in the seminiferous epithelium and the onset of spermatogenesis (Sharpe et al., 2003; Rey et al., 2009). This leads already to a weight-dependent positive androgen receptor expression and could explain the precocious positive reaction in our results, which is consistent to literature sources (Monet-Kuntz, Reviers & Terqui, 1984; De Gendt et al., 2004; Suriyaphol et al., 2020; Ge et al., 2020; Meroni et al., 2019).

## Conclusions

Our study demonstrates the potential for wild boar males to achieve considerably earlier puberty than so far supposed by hunting literature. Puberty occurred at nine months, the earliest being eight months of age, and with a dressed weight of 29 kg, with the lowest weight being 24 kg. Already as piglets, wild boar males showed a normal and completed spermatogenesis. Onset of sexual maturity occurred in piglets from ten months onwards and 30–35 kg of dressed weight, with 40 kg being the threshold dressed weight at which almost all wild boar became reproductively active. Thus, piglets under one year are of an age physiologically capable of fertilising females. Moreover, since piglets are usually born in spring, young males could match the end of the mating season in winter and target the heat of females of the same age, which also achieved sexual maturity during their first winter. Since we still do not know to which extent these individuals can take part in reproduction, more investigations are required in this field, for example, genetic and behavioural analyses. Considering the high prolificacy of wild boars already at a young age, precocious maturity may contribute to the steady population increase observed in Germany and the rest of Europe. Therefore, to achieve an effective management of wild boar populations, a reduction in wild boar numbers should focus also on piglets of both sexes, also in the context of African swine fever outbreaks.

## Supplemental Information

10.7717/peerj.11798/supp-1 Supplemental Information 1 Data on classification of wild boar puberty All collected samples and their information about collection date/location as well as age and weight data and a description of the histological observations performed on microscope for classification of the developmental stage/maturation for every animal. This data were used for statistical analysis.

## Additional Information and Declarations

### Field Study Permissions

The following information was supplied relating to field study approvals (i.e., approving body and any reference numbers): Permission for collection of samples was provided verbally by the following heads of operations: Mr. Helmut Beuke (Forest Commission Oerrel), Mr. Georg Deeken (Forest Commission Unterlüß) and Günther Graf von der Schulenburg (Graf von der Schulenburg).

### Data Availability

The following information was supplied regarding data availability: The raw data are available in the Supplemental File.
